# The impact of caregiver tele‐health support and education intervention on the relationship between caregiver grief and quality‐of‐life

**DOI:** 10.1002/alz.090419

**Published:** 2025-01-09

**Authors:** Sarah Gothard, Aimee Mooney, Emily Tupper, Allison Lindauer

**Affiliations:** ^1^ NIA‐Layton Aging & Alzheimer’s Disease Research Center, Portland, OR USA; ^2^ Oregon Health & Science University, Portland, OR USA; ^3^ Oregon Center for Aging & Technology (ORCATECH), Portland, OR USA; ^4^ Oregon Health and Science University, Portland, OR USA; ^5^ Oregon Health and Sciences University, Portland, OR USA; ^6^ OHSU School of Nursing, Portland, OR USA

## Abstract

**Background:**

Current research around caregiving for person’s with dementia (PWD) has historically emphasized caregiver burden. This leaves a gap of knowledge around other contributors to caregiving’s long‐term effects, including grief. The current analysis explores the relationship between caregiver grief and quality‐of‐life.

**Method:**

The Tele‐STELLA study is a multi‐component, telehealth‐based intervention that teaches caregivers strategies to manage upsetting behaviors related to dementia in 8 weekly, 1‐hour sessions with professionals. The caregivers in this analysis (n = 138) cared for a family member with moderate to late‐stage dementia. A multilinear analysis was used to explore the relationship between grief and quality‐of‐life for 138 caregivers of PWD. Caregivers were women (73.2%) and white (87.0%), with 82.6% reporting financial security. On average, caregivers were engaged in their roles for 4.64 years (SD 3.69); 87.0% of the PWDs were in the moderate stage of dementia. Pre and post questionnaires queried caregivers about frequency of problematic behaviors and their reactions to the behaviors, grief, and household quality‐of‐life.

**Result:**

Of the 138 caregivers who started the 8‐week intervention, 98 (71%) completed both pre/post self‐reported data. Scatterplot visualization demonstrated a linear negative relationship between grief/quality‐of‐life at both pre and post intervention timepoints. Controlling for sex and care recipient dementia severity, a multilinear regression was performed and showed, at alpha 0.05, a significant negative relationship between quality‐of‐life and grief (p‐value: 0.00, B1: ‐0.17) at the pre‐intervention timepoint. Post‐intervention analysis of the same model showed the magnitude of the B1 coefficient of grief changed to ‐0.22 and an increased significance (p‐value: 0.00).

**Conclusion:**

This analysis found that grief in dementia caregiving had a strong negative relationship with quality‐of‐life and that this relationship became stronger after intervention. This may be due to caregivers’ increased awareness of grief, or to the progressive nature of dementia over the 8‐week intervention period, but still highlights grief’s impact on caregiver quality‐of‐life. Future longitudinal studies will be necessary to evaluate if grief can serve as a predictor of quality‐of‐life and long‐term health outcomes for caregivers, and how best to leverage that knowledge for interventions in this population.

